# A Robust Expression and Purification Method for Production of *Sp*Cas9-GFP-MBP Fusion Protein for In Vitro Applications

**DOI:** 10.3390/mps5030044

**Published:** 2022-05-28

**Authors:** Andrea Luciana Fleitas, Mario Señorale, Sabina Vidal

**Affiliations:** 1 Laboratorio de Biología Molecular Vegetal, Instituto de Química Biológica, Facultad de Ciencias, Universidad de la República, Montevideo 11400, Uruguay; 2 Sección Bioquímica y Biología Molecular, Facultad de Ciencias, Universidad de la República, Montevideo 11400, Uruguay; msenorale@gmail.com

**Keywords:** CIRSPR/Cas9, expression, purification, immobilized metal affinity chromatography, ion exchange

## Abstract

Genome editing using the CRISPR/Cas9 system is one of the trendiest methodologies in the scientific community. Many genome editing approaches require recombinant *Streptococcus pyogenes* Cas9 (*Sp*Cas9) at some point during their application, for instance, for in vitro validation of single guide RNAs (SgRNAs) or for the DNA-free editing of genes of interest. Hereby, we provide a simple and detailed expression and purification protocol for *Sp*Cas9 as a protein fused to GFP and MBP. This protocol improves protein yield and simplifies the purification process by overcoming the frequently occurring obstacles such as plasmid loss, inconsistent protein expression levels, or inadequate protein binding to affinity resins. On average, this protocol yields 10 to 30 mg of purified, active, His6−MBP−*Sp*Cas9 NLS−GFP protein. The purity addressed through SDS-PAGE is > 80%.

## 1. Introduction

The CRISPR/Cas9 (Clustered Regularly Interspaced Short Palindromic Repeat-CRISPR associated protein 9) system is the most efficient, flexible, and broadly used technology for genome editing (GE). CRISPR/Cas9 based systems involve a powerful and continuously growing number of tools for a variety of applications, such as targeted DNA mutagenesis, transcriptional control of gene expression, nucleic acid dynamics imaging and epigenetic manipulation [1,2], as well as diagnostic assays [3]. 

While DNA-free methodologies have become popular for GE of animal cells [4,5], in plants, GE is frequently implemented using transgenesis to introduce a gene construct that allows the expression of Cas9 and the single guide RNAs (sgRNA). Stable transformed plants harboring the intended mutation can be subsequently self-pollinated or crossed to segregate the incorporated DNA construct to obtain transgene-free plants [6,7]. However, DNA-free strategies can be used in plants to guarantee the non-incorporation of foreign DNA into the genome. Instead of using DNA constructs, Cas9 can be directly delivered into the cells in the form of a protein or messenger RNA (mRNA), together with the in vitro synthesized sgRNAs [8,9,10,11,12,13,14]. 

Regardless of the method for delivering the editing machinery into the cells, most in vivo applications of CRISPR-based technologies require prior in vitro testing of sgRNA efficiency on target recognition using Cas9-sgRNA ribonucleoprotein (RNP). This is particularly important when working with plants, where editing performance can be highly affected by the transformation and tissue culture efficiencies of the plant species [6,7]. Even though Cas9 can be commercially acquired, the cost of this reagent can still be too high for emerging research groups or researchers working in low-income countries.

Although a number of protocols for *Streptococcus pyogenes* Cas9 expression and purification have been published (Table 1), we provide an improved step by step protocol that includes a number of modifications made over previously described ones. Most important modifications were performed at the bacterial expression stage but some key tips are also highlighted to prevent common pitfalls during purification.

In this protocol, *Sp*Cas9 is expressed as a protein fused to green fluorescent protein (GFP), and maltose binding protein (MBP). The GFP tag allows for the presence of the recombinant protein to be monitored throughout all the steps of the procedure, while MBP acts as a solubility enhancer tag.

While most other published protocols require three steps for the purification of *Sp*Cas9 [15,16,17,18,19,20,21], our protocol describes a two-step purification procedure that results in large quantities of active, MBP and GFP-fused *Sp*Cas9, which is suitable for in vitro applications. The entire purification protocol can be carried out within a single day, achieving a good compromise between purity and working time. In addition, our protocol resulted in a significant improvement of protein yield (~30 mg/L of bacterial culture), in comparison to yields reported for other previously described protocols, listed in Table 1 (~1.2 mg–~ 6 mg/L of bacterial culture) [15,22,23,24].

The protocol also describes the additional steps required for removal of MBP and the purification of *Sp*Cas9−GFP for in vivo GE applications. The GFP labeling of *Sp*Cas9 allows for the visible confirmation of the delivery of RNPs and the enrichment of the target cells after transfection of the eukaryotic cells, without compromising the activity of *Sp*Cas9 [18,25,26,27,28].

**Table 1:** Cas9 expression and purification protocols.

Plasmid	Reported Yield/L of Bacterial Culture	Reference
pMJ915 (MBP−Cas9)	1.77 mg	[15]
pET-NLS Cas9−6xHis	1.2 mg	[22]
pET-28b-Cas9−His (Cas9−His)	8.38 mg (best condition reported)	[23]
pET15 (Cas9−6xHis)	6 mg	[24]
His−MBP−Cas9 Cas9 D10A, H840A and D10A/H840A point mutants	NR *	[16,17]
PMJ922 (His−MBP−TEV−Cas9 NLS−GFP)	NR *	[18]
His−MBP−Cas9 Cas9 D10A, H840A and D10A/H840A point mutants	NR *	[19,20]
D10A/H840A dCas9	NR *	[21]
pET-Cas9 NLS−6xHis	NR *	[29]
pET-28b-Cas9−His	NR *	[30]
pET-28b-Cas9−His	NR *	[31]

* NR: not reported.

To our knowledge, this is the first report of a detailed Cas9 purification protocol that includes troubleshooting for each stage of the procedure. Moreover, alternative procedures are included in case not all of the listed equipment is available in the lab (Section 5).

## 2. Experimental Design

**Figure 1:**
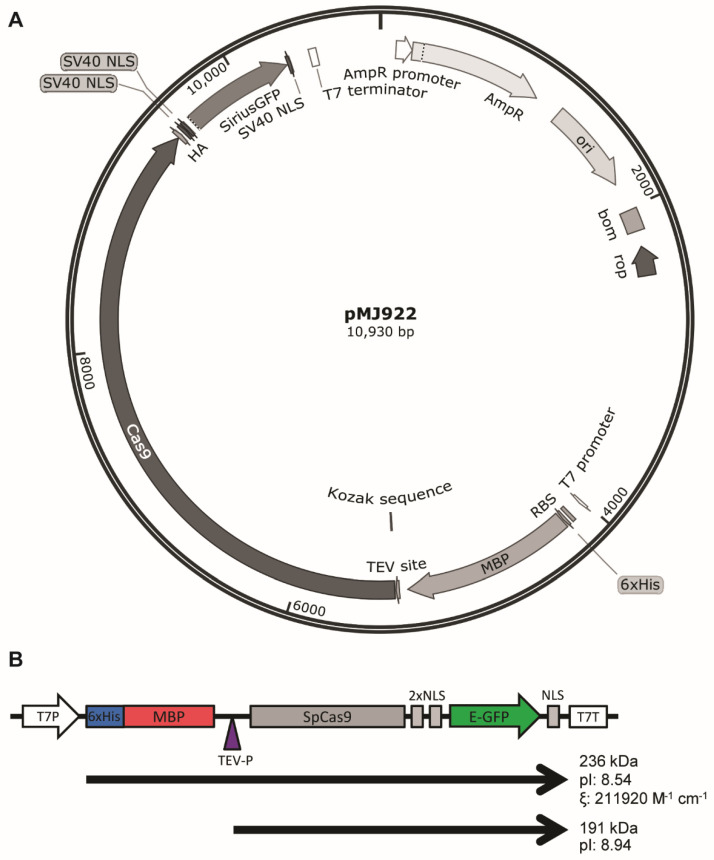
(**A**) PMJ922 map from Addgene. (**B**) 6xHis−MBP−*Sp*Cas9 NLS−GFP transcriptional unit. 6xHis−MBP can be removed by cleavage with TEV protease. Physicochemical properties for chimeric proteins *Sp*Cas9−GFP−MBP and *Sp*Cas9−GFP are indicated to the right.

The protein was expressed from plasmid pMJ922 [18] obtained from Addgene (Figure 1A). This plasmid allows the expression of *Sp*Cas9 fused in-frame with a C-terminal hemagglutinin (HA) epitope tag, a bipartite nuclear localization signal (NLS) sequence, a green fluorescent protein (GFP) polypeptide, and an additional monopartite NLS at the very C-terminus (*Sp*Cas9 NLS−GFP). *Sp*Cas9 NLS−GFP is expressed as a fusion protein with an N-terminal hexahistidine-maltose binding protein (His6−MBP) affinity tag (Figure 1) that can be removed by cleavage with Tobacco etch virus protease (TEV-P) during purification, depending on the desired application. The time needed to complete each stage is summarized in Table 2.

### 2.1. Materials

1. pMJ922 (Addgene, Cambridge, MA, USA; plasmid no. 78312).
2. *Escherichia coli* BL21 (DE3) pLysS (ThermoFisher Scientific, Waltham, MA, USA; Cat. no.: C602003).
3. TRIS (Duchefa Biochemie; Haarlem, The Netherlands; Cat. no.: T1501).
4. HEPES (Sigma-Aldrich, St. Louis, MO, USA; Cat. no.: H-3375).
5. Potassium chloride (Sigma-Aldrich; St. Louis, MO, USA; Cat. no.: P3911).
6. Magnesium chloride hexahydrate (Sigma-Aldrich; St. Louis, MO, USA; Cat. no.: M2670).
7. Sodium chloride (Sigma-Aldrich St. Louis, MO, USA; Cat. no.: S7653).
8. Tris(2-carboxyethyl)phosphine hydrochloride (TCEP; Sigma-Aldrich; St. Louis, MO, USA; Cat. no.: C4706).
9. DTT (Duchefa Biochemie; Haarlem, The Netherlands;Cat. no.: D1309).
10. Imidazole (Sigma-Aldrich; St. Louis, MO, USA; Cat. no.: I-2399).
11. Glycerol (Sigma-Aldrich; St. Louis, MO, USA; Cat. no.: G5516).
12. Benzonase (Pierce, Waltham, MA, USA; Cat. no. 88701).
13. Protease inhibitor tablets (Roche, Basel, Switzerland; Cat. no. 05892970001).
14. HiTrap IMAC FF (GE Healthcare; Chicago, IL, USA; Cat. no. 17092104).
15. PD-10 desalting columns (GE Healthcare; Cat. no. 17-0851-01).
16. HiTrap SP HP (GE Healthcare; Chicago, IL, USA; Cat. no. GE29-0513-24).
17. Polycarbonate bottles (Beckman, Brea, CA, USA; Cat. no. 355605).

### 2.2. Equipment

- Refrigerated orbital shaker (Labotech, Midrand, South Africa; Model: ZWY-211C).
- Magnetic stirrer (Thermolyne, Waltham, MA, USA).
- Sonicator (Benchmark Scientific, Sayreville, NJ, USA. Model: Pulse 150).
- Beckman J2HC refrigerated centrifuge (Beckman, Brea, CA, USA; Cat. no.: 8043-30-1105).
- Beckman JA-10 rotor (Beckman, Brea, CA, USA; Cat. no.: 369687).
- Beckman JA-20 rotor (Beckman, Brea, CA, USA; Cat. no.: 334831).
- ÄKTA FPLC system (GE Healthcare, Uppsala, Sweden; Cat. no.: 29-0598-78 AB).

## 3. Procedure

### 3.1. Protein Expression

**Figure 2:**
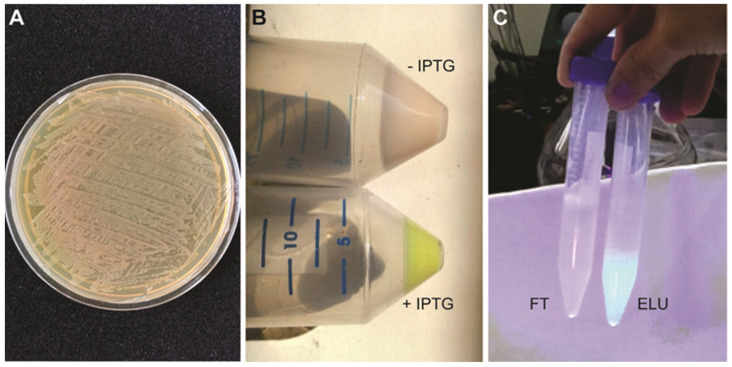
(**A**) Starting bacterial plate. (**B**) Greenish pellet obtained after induction with IPTG. (**C**) GFP fluorescence under UV light of different fractions during IMAC. FT: IMAC flow through; ELU: IMAC elution.

1. Transform pMJ922 into competent *E. coli* BL21 (DE3) pLysS. Isolate single colonies and cultivate them for 8 h in liquid LB containing 100 μg/mL ampicillin and 34 μg/mL chloramphenicol. Prepare stocks using 25% glycerol final concentration.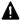 **CRITICAL STEP:** overgrowing bacterial cells may lead to plasmid loss. It is recommendable to check plasmid presence at this step either through PCR or miniprep.
2. Prepare starting plates for protein expression. Spread glycerol stock onto a solid LB plate containing 100 μg/mL ampicillin and 34 μg/mL chloramphenicol. Completely streak the plate to cover the entire plate with bacteria. Incubate at 37 °C overnight (Figure 2A).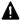 **CRITICAL STEP:** this plate is used in replacement of an overnight grown culture. Prepare two plates for this purpose.
3. Using a sterile spatula, scrape all the bacteria from a plate and resuspend the cells in 5 mL LB media. Add this suspension to a 2 L Erlenmeyer containing 500 mL TB media (24 g/L yeast extract, 20 g/L tryptone, 4 mL/L glycerol, 17 mM KH_2_PO_4_, 72 mM K_2_HPO_4_) supplemented with 200 μg/mL ampicillin and 34 μg/mL chloramphenicol. Incubate at 37 °C and 200 rpm until absorbance at 600 nm reach 0.6. Prepare two Erlenmeyers for this purpose.
4. Cool down the cultures at 4 °C for at least 30 min to arrest growth.
5. Supplement the cultures with ampicillin once again (to reach a final concentration of 400 μg/mL) and subsequently induce protein expression by adding 200 μM isopropyl-D-1-thiogalactopyranoside (IPTG). Cultivate for 16 h at 18 °C and 180 rpm.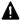 **CRITICAL STEP:** ampicillin reinforcement ensures that selection of the plasmid is maintained during overnight induction.
6. Harvest the cells in four 500 mL polycarbonate bottles (Beckman, Cat. no. 355605) by centrifugation at 3000× *g* for 20 min at 4 °C. Resuspend the cell pellet in 100 mL of the supernatant and transfer to two 50 mL Falcon tubes. Centrifuge at 3000× *g* for 20 min at 4 °C and weight the pellet.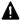 **CRITICAL STEP:** the pellet should be greenish due to the GFP expression (Figure 2B).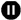 **PAUSE STEP:** pellets can be stored at −80 °C for several months until use.

### 3.2. Protein Purification

#### 3.2.1. Bacterial Lysis

1. Resuspend 3–6 g of cell pellet in IMAC buffer A (50 mM TRIS pH 7.5, 500 mM NaCl, 5 mM MgCl_2_) in a 5:1 buffer:pellet ratio. Perform an initial lysis step by three cycles of freezing/thawing the pellet at −80 °C. Transfer the suspension to a small beaker and add lysozyme to a final concentration of 0.1 mg/mL, 1 mM DTT final concentration, additional MgCl_2_, 1:500 *v*/*v* from 1 M stock solution, a protease inhibitor tablet (Roche, Cat. no. 05892970001) and 1 μL of benzonase (25 kU/μL, Pierce, Cat. 88701).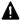 **CRITICAL STEP:** using benzonase instead of DNase greatly simplifies further purification steps. Benzonase digests all forms of nucleic acids, which can affect purity during IMAC and binding to the ion exchange resin if left undigested.
  Contamination with nucleic acids from *E. coli* can be problematic for Cas9 purification because these molecules can interfere with the binding capacity of Cas9 to the IEX resin due to PI alteration. This problem can be addressed by using RNase in addition to DNase or, alternatively, benzonase, which exhibits both DNase and RNase activity.
2. Incubate for 30 min on ice under very gentle stirring to avoid foam formation.
3. Perform 4 cycles of sonication using 40% amplitude pulses for 3 min, with pauses of 2 min in between them.
4. Clarify the suspension by centrifugation for 15 min at 16,000× *g* at 4 °C. Transfer the supernatant to a new tube and centrifuge again for 45 min at 16,600× *g* and 4 °C.
5. **OPTIONAL STEP:** filtrate through a 0.45 μm filter if the lysate is not totally transparent.

#### 3.2.2. IMAC

6. Load the soluble fraction into a 5 mL IMAC HiTrap FastFlow column (GE, Cat. 17092104), previously equilibrated with 10 column volumes (CV) of IMAC buffer A. Wash the column with at least 25 CV of IMAC buffer A. Elute resin-bound proteins with IMAC buffer B (50 mM TRIS pH 7.5, 250 mM NaCl, 5 mM MgCl_2_, 400 mM imidazole) in 0.5 mL fractions.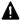 **CRITICAL STEP:** extensively wash unbound protein. More than 25 CV may be needed in some cases. Measure the absorbance of IMAC buffer A and wash until this value is reached again.

#### 3.2.3. Buffer Exchange in PD-10

7. Pool the protein containing fractions and perform buffer exchange in PD-10 column (GE, Cat. 17-0851-01) following the manufacturer’s instructions. Use ion exchange (IEX) buffer A (50 mM TRIS pH 7.5, 100 mM NaCl, 5 mM MgCl_2_) for equilibration. After elution, add DTT to 1 mM final concentration. For in vitro applications, continue in Section 3.2.4.
8. **OPTIONAL STEP:** For in vivo applications, it is recommended to remove the 6xHis−MBP tag by cleavage with TEV-P (see Section 3.2.7).

#### 3.2.4. IEX

9. Load the protein into the cation exchange SpHp HighTrap column (GE, Cat. GE29-0513-24) previously equilibrated with IEX buffer A. Wash the column with at least 25 CV of IEX buffer A. Elute the protein with a linear gradient of 0.1–1 M NaCl in 20 CV, collecting in fractions of 0.5 mL.
  **OPTIONAL STEP:** This step can be performed using a peristaltic pump, in which case elution is performed in a single step with IEX buffer B (50 mM TRIS pH 7.5, 1 M NaCl, 5 mM MgCl_2_). The purity obtained is not as good compared to the gradient elution, but the protein can still be used for in vitro cleavage assays.

#### 3.2.5. Buffer Exchange in PD-10

**Figure 3:**
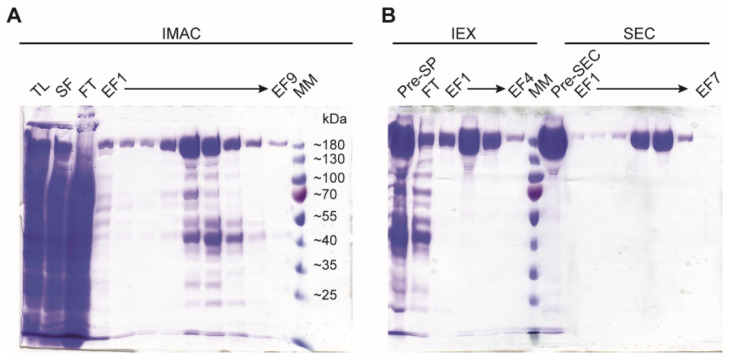
SDS-PAGE run on 10% acrylamide-bisacrylamide gel. (**A**) IMAC protein samples. (**B**) IEX and SEC protein samples. TL: total lysate; SF: soluble fraction; FT: flow through; EF: elution fraction; Pre-SP: pre SPHP high trap column; Pre-SEC: pre size exclusion; MM: molecular weight marker.

10. Pool the protein containing fractions and perform buffer exchange in a PD-10 column equilibrated in storage buffer (30 mM HEPES pH 7.5, 200 mM NaCl, 2 mM MgCl_2_, and 1 mM TCEP).
11. **OPTIONAL STEP**: If greater purity is needed, use a Superdex 200 16/600 size exclusion column (GE Healthcare), equilibrated in storage buffer for the final polishing step (Figure 3).

#### 3.2.6. SDS-PAGE

12. Check the purity of the purified protein by SDS-PAGE in 10% acrylamide-bisacrylamide (29:1) gel (Figure 3).

#### 3.2.7. Removal of His−MBP Tag for In Vivo Applications (Optional Step 15, Expanded)

If free *Sp*Cas9−GFP is needed, buffer exchange with a desalting column can be substituted by dialysis against IEX buffer A in the presence of TEV-P. Collect the protein eluted from IMAC (Section 3.2.2) and add DTT to 1 mM final concentration, EDTA to 1 mM final concentration, and 1 mg of TEV-P for every mg of protein.

Place the protein solution in dialysis tubing with a cutoff >12 kDa. Perform dialysis overnight at 4 °C against buffer IEX A. Place the dialysis tubing inside a 500 mL graduated cylinder with constant stirring for this purpose.

To separate free *Sp*Cas9−GFP from His−MBP, perform a second IMAC (IMAC2) using the same conditions as the first IMAC (Section 3.2.2). His−MBP and *Sp*Cas9−GFP bound to MBP will be retained in the IMAC column, while free *Sp*Cas9−GFP will be present in the flow through (Figure S2).

Buffer exchange *Sp*Cas9−GFP obtained from this step as described in Section 3.2.3 and proceed with Section 3.2.4.

#### 3.2.8. Protein Storage

13. Quantify the protein concentration using theoretical ξ_280_ = 211,920 M^−1^ cm^−1^.
14. Store the protein at a final concentration > 1 mg/mL (usually the protein concentration is higher than 1 mg/mL after buffer exchange). Prepare single use aliquots and flash freeze in liquid nitrogen. Store at −80 °C. The protein remains active for over a year.

### 3.3. In Vitro Nuclease Activity Assay

**Figure 4:**
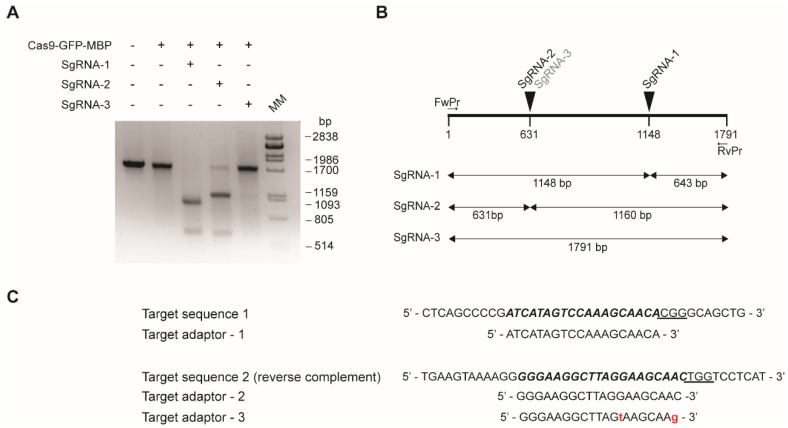
In vitro cleavage assay. (**A**) Separation of cleaved DNA fragments by electrophoresis in 1% agarose gel. A 1791 bp DNA fragment corresponding to Glyma.06G072000 gene was PCR amplified from soybean genomic DNA using FwPr and RvPr primers. Two specific sgRNAs (sgRNA-1 and sgRNA-2) were designed to target different regions of the gene. An additional sgRNA (sgRNA-3), containing two mismatches with sgRNA-2, was designed to assess RNP specificity. The genomic sequence, sgRNA target sequences and primer sequences are listed in Figure S1, Supplementary Materials. The target DNA sequence was incubated with *Sp*Cas9−GFP−MBP in the presence (+) or absence (−) of sgRNAs. DNA cleavage resulted in sharp bands. The size difference between large fragments slightly deviates from what is expected, which could be explained by electrophoretic artifact. A weak band corresponding to the uncleaved pcr product can be observed in the gel when sgRNA2 was used for target recognition. MM: λ DNA digested with *Pst*I restriction enzyme. (**B**) Schematic representation of the target genomic sequence, the position of the sgRNAs target sequences and the expected sizes of the DNA fragments after cleavage. (**C**) Target sequences and sgRNA spacer sequences (target adaptors) are shown to the right. PAM sequences are underlined, and mismatches are in red.

1. Prepare 50 μL of reaction mixture in 1X Cas9 buffer (0.2 mM HEPES pH 7.5, 1.5 mM KCl, 0.1 mM MgCl_2_, 10 μM EDTA, 50 μM DTT freshly added) as indicated in Table 3.
2. Allow the reaction to equilibrate for 15 min at room temperature. Add 300 ng of the PCR product, briefly vortex and spin. Incubate at 37 °C for 2 h.
3. Add 1 μL of 20 mg/mL proteinase K and incubate for 10 min at 37 °C. Analyze in agarose gel. The agarose percentage should be according to the expected DNA fragment sizes after cleavage. An example of a typical activity assay is shown in Figure 4.

## 4. Expected Results

On average, this protocol yields 10 to 30 mg of purified, active, His6−MBP−*Sp*Cas9 NLS−GFP protein (~10 mL at 1–3 mg/mL). The purity addressed through SDS-PAGE is >80%.

## 5. Protocol Alternatives and Troubleshooting

Protocol alternatives can be found in Table 4 and troubleshooting in Table 5.

## Figures and Tables

**Table 2:** Experimental design.

Activity	Step	Day 1	Day 2	Day 3	Day 4	Day 5	Day 6
**Bacterial transformation**	1	ON					
**Glycerol preparation**	1		8 h				
**Preparation of starting plate**	2			ON			
**Bacterial growth**	3–5				3–4 h		
**Induction**	5				ON		
**Harvest**	6–7					1 h	
**Lysis and clarification**	8–12						2.5 h
**IMAC**	13						1.5 h
**Buffer exchange**	14						30 min
**IEX**	16						1.5 h
**Buffer exchange**	17						30 min
**SDS-PAGE**	19						1.5 h
**Storage**	20–21						30 min

**Table 3:** Nuclease activity reaction.

	Volume (μL)
H_2_O _DEPC_	To 50 μL
Buffer 10X Cas9 + DTT	5 μL
His6−MBP−*Sp*Cas9 NLS−GFP	X μL (4 μg)
SgRNA	X μL (1120 ng)

**Table 4:** Protocol alternatives.

Step	Suggested Procedure	Alternative
**3.2.2 IMAC**	IMAC HiTrap FastFlow column (GE, Cat. no.: 17092104) connected to ÄKTA FPLC system (GE, Cat. no.: 29-0598-78 AB)	**A.** Perform IMAC with the column connected to a peristaltic pump. Follow absorbance at 280 nm using a spectrophotometer. Equilibrate the IMAC column with 10 CV on IMAC buffer A and measure the buffer absorbance before loading your protein sample. Make sure that this value is reached again after extensively washing bound protein. **B.** Perform batch incubation with IMAC resin. Follow the absorbance at 280 nm using a spectrophotometer. Equilibrate the resin in IMAC buffer A and measure the buffer absorbance before loading your protein sample. Make sure that this value is reached again after extensively washing bound protein.
**3.2.3 Buffer exchange**	Perform buffer exchange in PD-10 column (GE, Cat. 17-0851-01) into IEX buffer A.	**A.** Buffer exchange the protein using centrifugal filter units with a cutoff >50 kDa and perform several dilution/concentration steps. Use IEX buffer A as the diluting solution.
**3.2.4 IEX**	Cation exchange SpHp HighTrap column (GE, Cat. GE29-0513-24) connected to ÄKTA FPLC system (GE, Cat. no.: 29-0598-78 AB)	**A.** Perform IEX with the column connected to a peristaltic pump. Follow the absorbance at 280 nm using a spectrophotometer. Equilibrate the IMAC column with 10 CV on IEX buffer A and measure the buffer absorbance before loading your protein sample. Make sure that this value is reached again after extensively washing bound protein. Perform elution in a single step with 1 M NaCl concentration or in several steps with increasing NaCl concentrations.
**3.2.5 Buffer exchange**	Perform buffer exchange in the PD-10 column (GE, Cat. 17-0851-01) into storage buffer.	**A.** Buffer exchange the protein using centrifugal filter units with a cutoff >50 kDa and perform several dilution/concentration steps. Use storage buffer as the diluting solution. **B.** Use a Superdex 200 16/600 size exclusion column (GE Healthcare), equilibrated in storage buffer for the final polishing step.

**Table 5:** Troubleshooting.

Problem	Possible Explanation	Solution
**No protein expression**	Plasmid loss	Retransform BL21 Rosetta or use another aliquot from your bacterial stock. Check plasmid presence in your bacterial stock.
**High protein contamination during IMAC**	Incomplete wash of nonspecific proteins	Do not elute protein until buffer absorbance from the washing fraction reaches the starting value. Try performing a washing step with IMAC buffer A containing 5 mM imidazole. This can lead to some Cas9 washing as well.
**No protein binding to IEX column**	Nucleic acid contamination.	Treat the protein fraction with benzonase.

## Data Availability

Not applicable.

## Supplementary Materials

The following supporting information can be downloaded at: https://www.mdpi.com/article/10.3390/mps5030044/s1, Figure S1: Sequence information for RNP activity assays; Figure S2: MBP removal from MBP-*Sp*Cas9-GFP.
