# Supplementary material for: A Robust Expression and Purification Method for Production of SpCas9-GFP-MBP Fusion Protein for In Vitro Applications
Source: Methods Protoc. 2022 May 28;5(3):44. doi: 10.3390/mps5030044 (PMC9228339; doi:10.3390/mps5030044)
Supplement: Supplementary file 1 [file mps-05-00044-s001.zip › mps-1650933-supplementary.pdf]

>Glyma.06G072000

FwPr →

CACATATCACCGAAAAATAAATTAATTTAAAAAATTAGATGATCAAATTAAAATTAAAAAAATTAATTGATGAAATTAAATCG
ATTTTAAAAAATAAAAAATAAAATGAAAGTTACGGAATTGAAATATCTAAATCCAGAAGAAGAGTGAAGGGGAAACTTGTTTA
ATCGGTTAGTTTCCCTATTACGAAATTGTTTGACATAATTTTTTCAATGAATCCTCTTGTTTGAAACTTACATAAGTTTGATC
GAGGAAGTCCAAATTTTCATCCAAGATGCATGGAGAGACTGTGACAAGCGTGTCCTCTACTGGGCAACGCGAGATTGCTGCAA
ACACGGCAAGCAATAGGTAGCACCAAAAACAAGATGCTAAACTCAACCAATTAATTAAAAGTAAATAATGGTGGCTTTGCCAT
TTGGAATACGCCTCTTCCACTTGTCCAAACAATAACTCCCACTTTTTTCGATCTCCTAATTACTATTTTACATTTCAAGCTTT
TCTGCTTCCTCATATCTCTCTATATATTGCTTCCTTTAGTTCTAAGTCAAGACTTTTATAATTAAGGTAAGAGGGTCATCGTG
GAAGGATTTATAAATTTTCTCAACTCAACTCTCACCATGAGGACCA**GTTGCTTCCTAAGCCTTCCC**CCTTTTACTTCAAACCA
ACCTTCCATTCCCCCAAAACATCCTCAACTTTCATCGTAAGTTTTCATCTTTGTTTCTACATACATATATTTTCTTTTAACTT
GTTGGGTCACAATTTACATGTCATTTATGTGACTCATGTCACTAACATTACCGTGTGCGTTGTAATAAAGGGTGAAGAACGAA
GCATGTTGGAAGAGGCAATGCGTTGTGATGGGAGTGGCATCCATTATTGGACTAGAAATGTGCAATTCAGTGGCAATGGCCCA
CGAAGCAATTGAAATCAAGACCATGCCATTTAGTAACCAAGTAGTATCAAATAGCAATTCTTACGGTGGCGCCAAATGGAGCG
AGAAAAGAATGTGCCCACCTTGGCAAGGCAATTCGCTCGAAACGATCGTGCCGGAGAATCTTCCCCGGCCGTCGGCACGGCGG
AGATACGAGGCTGTTCGTTCCTCCTCCAAGACTGCGCCGCCGCTCTCCGCCCCG**ATCATAGTCCAAAGCAACA**AGGGCAGTTG
CTTCTCCATGTGAAAAATACTAAATCAAATTAAATTTACTTGTACATGATTATTGTTAATTTAATTGCTTGGCCTTTCACACA
AACTTTTAATTTCATAATCATATCCATGTGTCAAGTTTTTATTTCTTGGATGAAAACTTCAAAAATATCTAACGAATAATTGT
ATGTAATGAATAGTACTAGTACTGGATTGGATTTATGCAGTTTTAGTGATTGTAGTACATGTAGTACAAATTCTAATTCGTTA
CCTATCTTAAACCATGCACAAGCACAAACATTGTACTACACTGCTTCCCATCTTTTCTTGTTGTTTCGTTTTTGTTTGCTTTC
TGAAGCCCATTAGTTTTATAGGCCTGCGGCCCTATTCGCTTTCTATTGGGCTGGTTCATCAGGCCATTATCGCTAACGCACAT
TCCTCGTTAAATTGTTGCTATCTGTTCATCGTCCCCGCCAATTTTTATGCGATTTATATTTAAATTTTTAATTTAAGAAAAAA
GAATTTAAAACATTAAATTACGTGTTATATAAATATTGATGGGACTATGAAGTCCACAGAATCACTTAATAATTTCTCCCATG
CAAAGATAATTCTTGAATCACATACC**AACCAGAGCTATTTTGTCTCCG** ← **RvPr**

Figure S1. Sequence information for RNP activity assays. sgRNA target sequences and primer sequences； 5′-untranslated region; exons; 3′-untranslated region. FwPr: forward primer; RvPr: Reverse primer. SgRNA-1 and sgRNA-2 target regions are boxed.

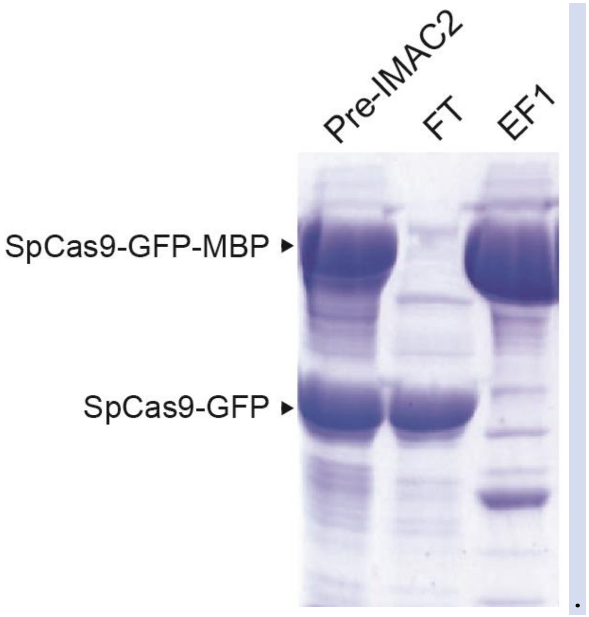

Figure S2. MBP removal from MBP-*Sp*Cas9-GFP.

Eluted MBP-*Sp*Cas9-GFP from IMAC was dialyzed overnight at 4°C with constant stirring against buffer A IEX using a dialysis tubing with a cutoff > 12 kDa. One 1mg TEV-P was used for every mg of protein, in the presence of 1 mM DTT and 1 mM EDTA. A sample of the protein mixture was loaded in the gel, showing partial digestion (pre-IMAC2). A second IMAC (IMAC2), performed under the same conditions as IMAC1 was done to recover *Sp*Cas9-GFP, which can be observed in the flow though (FT). MBP-*Sp*Cas9-GFP was retained in the IMAC column and eluted with imidazole (EF1: eluted fraction 1). SDS-PAGE was run in an 8 % acrylamide-bisacrylamide gel.
